# Cochlear Implantation in Adults 65 Years and Older With Single-sided Deafness and Asymmetric Hearing Loss

**DOI:** 10.1097/ONO.0000000000000094

**Published:** 2026-07-09

**Authors:** Daniel M. Zeitler, Nathalie Chouery, Seth R. Schwartz, Maura K. Cosetti

**Affiliations:** 1 Department of Otolaryngology-Head and Neck Surgery, Virginia Mason Franciscan Health, Seattle, Washington; 2 Department of Otolaryngology-Head and Neck Surgery, Icahn School of Medicine at Mount Sinai, New York, New York.

**Keywords:** Asymmetric hearing loss, Cochlear implant, Medicare, Older adults, Single-sided deafness

## Abstract

**Background:**

Cochlear implantation has proven success as a treatment for single sided deafness (SSD) and asymmetric hearing loss (AHL) in children and adults. However, in adults 65 years and older, cochlear implantation for the treatment of SSD and AHL has been difficulty to study given the restrictions in insurance coverage for cochlear implants (CI) due to current Medicare guidelines.

**Objective:**

This study aimed to evaluate outcomes in patients 65 years old and older who underwent cochlear implantation for SSD and AHL to provide data supporting a change in CI candidacy indications for Medicare beneficiaries.

**Methods:**

A cohort of CI recipients over the age of 65 years with either SSD (4-frequency pure tone average [4fPTA] ≥90 dB in the implanted ear and ≤30 dB in the better hearing ear) or AHL (interaural difference in 4fPTA ≥15 dB HL, better hearing ear 4fPTA >30 dB HL and ≤70 dB HL) was identified from a single tertiary-level CI center.

**Results:**

Forty CI recipients ≥65 years (9 SSD; 31 AHL) were identified. There were significant improvements (percentage point change, ∆%) demonstrated with CI use on consonant-nucleus-consonant (CNC) scores from baseline to 3 timepoints in both SSD (3 m, +38 ∆%; 6 m, +40 Δ%; 12 m, +47 Δ%) and AHL subjects (3 m, 40 Δ%; 6 m, +41 Δ%; 12 m, +42 Δ%). Significant improvements were also seen on AzBio sentences in quiet in both SSD (3 m, +67 Δ%; 6 m, +45 Δ%; 12 m, +68 Δ%) and AHL subjects (3 m, +44 Δ%; 6 m, +57 Δ%; 12 m, +54 Δ%). The status of the contralateral ear accounted for little variance in CNC improvement at 6 or 12 months. Moderate positive associations between device usage and CNC word and AzBio sentence testing were observed at 6 months.

**Conclusions:**

Outcomes in adults ≥65 years old with SSD and AHL who undergo CI support the use of implantation in Medicare beneficiaries and provide further evidence for ear-specific candidacy criteria.

Cochlear implants (CIs) were first approved by the Food and Drug Administration (FDA) for widespread use in adults in 1984. Since that time, data supporting the auditory benefits of cochlear implantation in adults with postlingual onset of sensorineural hearing loss (SNHL) have been well established and include significant gains in audibility and speech understanding not attainable with traditional hearing aid amplification. Candidacy for cochlear implantation has also evolved, reflecting advances in both device technology and a greater understanding of auditory health. In 1984, Medicare coverage for CIs was limited to adults with bilateral profound SNHL who could no longer benefit from properly fit hearing aids. Coverage indications during these early years required patients to demonstrate they received minimal benefit from hearing aids in both ears, a criterion based on limited clinical data at the time (1). As outcomes improved and CI device indications expanded, Medicare broadened its coverage in 2005 to include individuals with severe-to-profound hearing loss in both ears who demonstrated minimal speech recognition ability with conventional hearing aids (1). This change recognized CIs could help a larger group of people who were unable to achieve functional hearing with hearing aids, but the focus remained on bilateral hearing loss. With advances in the technological, clinical, and surgical aspects of cochlear implantation, candidacy indications continued to evolve. In 2022, following accumulation of large-scale evidence demonstrating benefit to adults with less than or equal to 60% correct on tests of open-set sentence recognition, the national coverage determination was amended and remains the current Medicare eligibility standard (2).

Currently, Medicare beneficiaries are covered for a CI only when they have bilateral moderate-to-profound hearing loss with limited benefit from amplification, as defined by test scores of ≤60% correct in the best-aided listening condition on recorded tests of open-set sentence cognition (3). At present, adults ≥65 years with severe-to-profound unilateral SNHL and normal or near normal hearing in the contralateral ear (also referred to as single-sided deafness, SSD) or those with asymmetric hearing loss (AHL) are ineligible for cochlear implantation under current Medicare indications.

Outside of Medicare, indications for cochlear implantation in patients with SSD and AHL have rapidly evolved. Robust data supporting the audiological and quality of life benefits of CI in the SSD and AHL patient populations spurred changes in FDA labeling for cochlear implantation beginning in 2019. The FDA approved the MED-EL CI system (MED-EL GmbH, Innsbruck, Austria), for SSD and AHL in 2019 and the Cochlear Nucleus CI system (Cochlear Americas, Centennial, CO) in 2022 for adults and children over 5 years of age (4). Following these changes, many private healthcare insurers, as well as the Veterans Administration and Department of Defense, included CI as a rehabilitative option for patients with SSD and AHL (5).

Progression of hearing loss over time is often asymmetric, affecting each ear independently. Many older adults experience asymmetric decline in their hearing over time, while others encounter sudden onset of unilateral severe-profound SNHL (6,7). Evidence supporting the benefit of CI in adults with SSD and AHL (including data used to support the aforementioned change in FDA labeling) has included some older individuals; however, given the inherent reimbursement challenges in this group, there is very limited data focused exclusively on Medicare beneficiaries (5,8). The present study aims to investigate the safety, efficacy, and ear-specific outcomes following CI in a population of adults ≥65 years of age with SSD and AHL.

## MATERIALS AND METHODS

### Patient Cohort

Adults ≥65 years old with SSD or AHL who underwent cochlear implantation at a single tertiary-level CI center between 2011 and 2024 were identified. SSD was defined as a 4-frequency pure tone average (4fPTA: 0.5, 1, 2, and 4 kHz) ≤30 dB HL in the better hearing nonimplanted ear and a 4fPTA ≥90 dB HL in the ear to be implanted. AHL was defined as an interaural difference in 4fPTA ≥15 dB HL with a better hearing ear 4fPTA >30 dB HL and ≤70 dB HL and a 4fPTA ≥70 dB HL in the ear to be implanted. For thresholds where no response was obtained, the following typical output limits of an audiometer were used to calculate the 4fPTA: 110 dB HL for 500 Hz and 120 dB HL for 1000, 2000, and 4000 Hz.

Patients with hearing loss due to vestibular schwannoma, those with radiologic imaging confirmation of cochlear malformations, individuals who underwent concurrent otologic procedures (eg, labyrinthectomy), or revision CI surgery were excluded. An institutional cloud-based CI data repository (REDCap) was queried. Identified patient medical records were reviewed, and data collected on patient demographics, including sex, age, race/ethnicity (self-identified), as well as preoperative hearing level, duration and etiology of hearing loss, perioperative complications, auditory performance metrics, and device usage (hours/day). Institutional Review Board (IRB) approval was obtained before data collection (IRB approval numbers 24-00149 Mount Sinai; 16-112 Virginia Mason Franciscan Health).

### Procedures

Pre- and post-implantation audiologic assessment, including aided and unaided pure tone thresholds and speech perception testing, was performed with the patient seated in a sound booth. Preoperatively, patients were tested in the best-aided condition. Patients with SSD were tested with a hearing aid or a transcranial rerouting device (eg, contralateral routing of sound, CROS aid) on the ear to be implanted, while the nontest ear was masked with 60 dB HL noise via earphone. Adults with AHL were tested with best-fit aids binaurally (using real ear measures according to NAL standards). Ear-specific and binaural tests of speech perception, including consonant-nucleus-consonant (CNC) monosyllabic words and AzBio sentences, were performed in quiet (CNC and AzBio quiet) at 60 dBA (9,10).

Postoperatively, ear-specific testing was performed using the same masking via earphone protocol or by direct connection to the CI (depending on audiologist preference). Binaural testing was performed in the best-aided condition, including a hearing aid in the nonimplanted ear for patients with AHL. Changes in speech perception testing scores from baseline evaluation to the postoperative time points (3, 6, and 12 months) were reported in percentage point change (Δ%) with positive scores representing improvement and negative scores representing worsening.

### Statistical Analysis

Statistical analyses were conducted using Microsoft Excel and R. Descriptive statistics were used to summarize patient characteristics. Pre- and postimplantation outcomes were compared using paired *t*-tests or nonparametric equivalents where appropriate. Categorical variables were assessed using Chi-square tests. A *P* value of <0.05 was considered statistically significant. Given the modest sample size and number of comparisons performed, findings were interpreted with emphasis on effect sizes and 95% confidence intervals rather than *P* values alone.

## RESULTS

### Participant Demographics and Clinical Characteristics

Clinical and demographic characteristics are presented separately for the SSD and AHL groups in Tables 1 and 2, respectively. Relevant variables include age at the time of implantation, insurance coverage type, and etiology of hearing loss.

**TABLE 1.** *Demographic and clinical characteristics of the SSD group (n = 9*)

Variable	N, %	M (SD)	Range
Age at implantation (yrs)		69 (3.35)	65–76
Preoperative 4f PTA (dB HL)			
Better ear		17.9 (5.04)	11.2–25
Ear to be implanted		104.17 (22.65)	70–125
Pre-Op aided CNC^*a*^ (%)		2.67 (7.28)	2.00–22.0
Pre-Op aided AzBio quiet^*a*^ (%)		2.67 (7.28)	2.00–22.0
Insurance coverage			
Medicare HMO	5, 55.6%		
Medicare^*b*^	3, 33.3%		
Commercial	1, 11.1%		
Age at SNHL diagnosis (yrs)		64.9 (3.86)	60–70
Etiology			
SSNHL	8, 88.9%		
Meniere’s disease	1, 11.1%		
Duration of SNHL, any severity (yrs)		3.87 (3.85)	0.30–10
Use of amplification (yrs)		2.85 (4.00)	0.03–10

^*a*^Ear to be implanted.

^*b*^Three patients received CI as research study participants.

4f PTA indicates 4-frequency pure-tone average; AzBio, Arizona Biological Sentences; CNC, consonant-nucleus-consonant; HMO, health maintenance organization; SD, standard deviation; SNHL, sensorineural hearing loss; SSNHL, sudden SNHL; yrs, years.

**TABLE 2.** *Demographic and clinical characteristics of the AHL group (n = 31*)

Variable	N, %	M (SD)	Range
Age at implantation (yrs)		75.2 (7.27)	65–91
Preoperative 4f PTA (dB HL)			
Better ear		55.3 (11.3)	31.2–70.0
Ear to be implanted		95.0 (13.7)	76.2–125.0
Pre-Op aided CNC^*a*^ (%)		7.61 (11.1)	0.00–36.0
Pre-Op aided AzBio quiet^*a*^ (%)		10.13 (15.72)	2.00–22.0
Insurance Coverage			
Medicare^*a*^	17, 54.8%		
Medicare HMO	11, 35.5%		
Commercial	3, 9.7%		
Age at SNHL diagnosis (yrs)		55.3 (17)	20–84
Etiology			
SSNHL	18, 58.06%		
Meniere’s disease	4, 12.90%		
Progressive SNHL	2, 6.45%		
Unknown	2, 6.45%		
Autoimmune	3.23%		
Duration of SNHL, any severity (yrs)		19.2 (16.0)	0.25–62
Use of amplification (yrs)		11.9 (12.4)	0.25–50

Three patients received CIs as research study participants.

^*a*^Ear to be implanted.

4f PTA indicates 4-frequency pure-tone average; AzBio, Arizona Biological Sentences; CNC, consonant-nucleus-consonant; HMO, health maintenance organization; SD, standard deviation; SNHL, sensorineural hearing loss; SSNHL, sudden SNHL; yrs, years.

As expected, there was a significant difference in better-ear 4fPTA between the SSD and AHL groups (Mann–Whitney U, U = 0, Z = −4.51, *P* < 0.00001, r = 0.71). In contrast, no significant difference was observed in the 4fPTA of the ear to be implanted (U = 178, Z = 1.23, *P* = 0.218, r = 0.19), suggesting only a small, nonsignificant difference in thresholds. Additionally, preoperative CNC scores (2.6%, 7.6%) and AzBio in quiet scores (2.6%, 10.1%) in the ear to be implanted did not differ significantly between the SSD and AHL groups (CNC: U = 87.5, Z = −1.79, *P* = 0.074, r = 0.28; AzBio: U = 69, Z = −1.01, *P* = 0.312, r = 0.17), with both comparisons showing small effect sizes.

The mean age for the entire cohort is 73.8 years (range, 65–91 years). Statistically significant group differences were observed for both age and duration of hearing loss. Mean age was significantly higher in the AHL group (75.2 years) compared with the SSD group (69 years), with a mean difference of 6.2 years (95% confidence interval, 2.8-9.7 years; U = 60.5, Z = −2.55, *P* = 0.011), with a moderate effect size (r = 0.40) and a common language effect size of 0.22. Similarly, the duration of hearing loss was significantly longer in the AHL group (19.2 years vs 3.9 years), with a mean difference of 15.4 years (95% confidence interval, 8.8-22.0 years; U = 36, Z = −3.23, *P* = 0.0012), with a large effect size (r = 0.52) and a common language effect size of 0.14. Age at hearing loss diagnosis was significantly higher in the SSD group compared with the AHL group, with a mean difference of 9.5 years (95% confidence interval, 2.6-16.5 years; t = 2.80, *P* = 0.008).

### Surgical Safety

All 40 subjects underwent successful cochlear implantation with complete electrode insertion. There were no intraoperative complications across the cohort. Thirty-nine of 40 subjects were discharged on the day of surgery; the remaining individual experienced an episode of atrial fibrillation that spontaneously converted to normal sinus rhythm and was discharged on postoperative day one without further complication. Postoperative complications occurred in 5 participants (12.5%) and included 2 cases of dizziness or imbalance (treated with oral medication, self-resolved), one case of transient taste disturbance, and one emergency room admission for altered mental status. Additionally, one patient experienced device failure (Advanced Bionics HiRes Ultra V1) necessitating revision surgery and reimplantation, which was performed without incident. Overall, the surgical procedure was well tolerated with a low rate of adverse events, consistent with previously reported safety profiles in adult CI recipients.

### Speech Perception Outcomes and Device Use

Tables 3 and 4 summarize ear-specific (CI alone) speech perception performance and device use over time.

**TABLE 3.** *Postoperative speech perception outcomes and device use for the SSD group (N = 9*)

Ear-specific outcome for CI alone	N	M (SD)	Range
Change in CNC Score (Δ%)			
Baseline to 3 m	5	+38.0 (23.5)	10.0–64.0
Baseline to 6 m	8	+39.5 (20.4)	0.00–58.0
Baseline to 12 m	5	+47.2 (7.29)	42.0–56.0
Change in AzBio in Quiet Score (Δ%)			
Baseline to 1–3 m	3	+67.3 (16.2)	50.0–82.0
Baseline to 6 m	5	+50.6 (29.7)	0.00–77.0
Baseline to 12 m	3	+68.3 (3.06)	65.0–69.0
Device use (average hrs/day)			
1–2-m post-CI	7	10.6 (3.70)	5.30–14.3
3-m post-CI	5	9.54 (3.54)	5.10–14.8
6-m post-CI	8	9.74 (4.63)	0.70–13.5
12-m post-CI	5	8.46 (2.97)	5.60–12.6

AzBio indicates Arizona Biological Sentences; CI, cochlear implant; CNC, consonant-nucleus-consonant; hrs, hours; m, months.

**TABLE 4.** *Postoperative speech perception outcomes and device use for the AHL group (N = 31*)

Ear-specific outcome for CI alone	N	M (SD)	Range
Change in CNC (Δ%)			
Baseline to 3 m	18	+40.4 (18.9)	16.0–86.0
Baseline to 6 m	21	+41.1 (18.9)	2.00–74.0
Baseline to 12 m	12	+42.2 (16.8)	16.0–78.0
Change in AzBio(Δ%)			
Baseline to 1–3 m	16	+43.7 (21.5)	5.00–76.0
Baseline to 6 m	20	+57.5 (24.9)	10.0–90.0
Baseline to 12 m	11	+54.45 (22.93)	29.0–87.0
Device use (average hrs/day)			
1–2-m post-CI	12	8.77 (3.09)	3.60–13.3
3-m post-CI	9	10.28 (2.97)	5.60–14.2
6-m post-CI	8	11.50 (3.49)	6.70–16.6
12-m post-CI	10	10.57 (2.59)	6.40–13.8

AzBio indicates Arizona Biological Sentences; CI, cochlear implant; CNC, consonant-nucleus-consonant; hrs, hours; m, months.

There were no statistically significant differences in CNC score improvements between the SSD and AHL groups at 3 months (U = 43, Z = −0.11, *P* = 0.911, r = 0.02), 6 months (U = 89.5, Z = 0.24, *P* = 0.807, r = 0.05), or 12 months postoperatively (U = 37.5, Z = 0.74, *P* = 0.459, r = 0.18). At 12 months, the mean between-group difference in CNC improvement was 5.0 percentage points (95% confidence interval, −7.4 to 17.5 percentage points). Effect sizes were small across all time points, indicating minimal between-group differences in word recognition gains. Similarly, no statistically significant between-group differences (SSD vs AHL) were found for AzBio sentence scores in quiet from preoperative to postoperative time points (pre- to 3 months: *P* = 0.74, effect size = 0.068; pre- to 6 months: *P* = 0.74, effect size = 0.068; pre- to 12 months: *P* = 0.53, effect size = 0.17). At 12 months, the mean between-group difference in AzBio improvement was 13.9 percentage points (95% confidence interval, −1.8 to 29.6 percentage points).

**FIG. 1.**
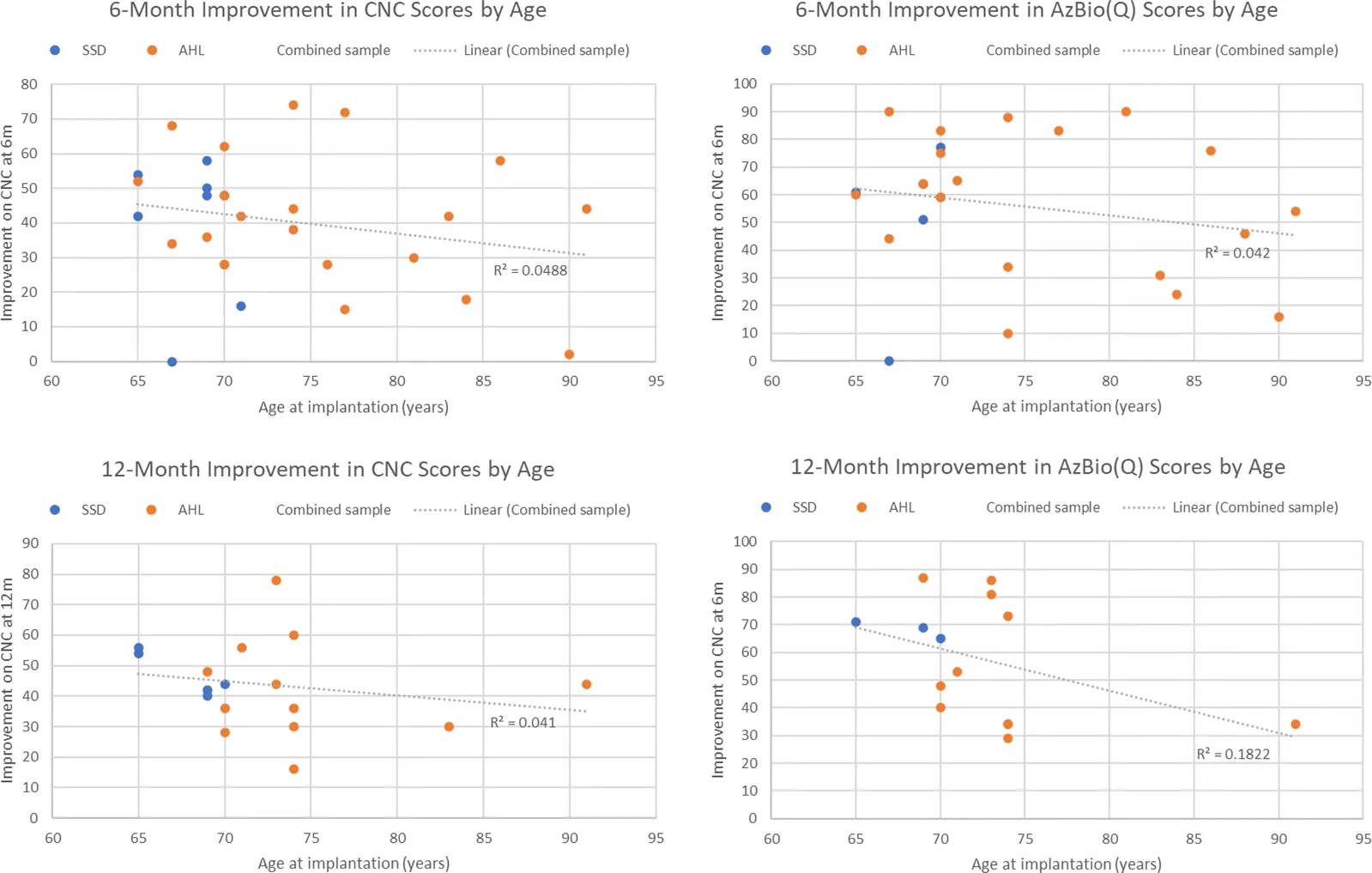
Postoperative improvement in CNC and AzBio(Q) scores by age. CNC indicates consonant-nucleus-consonant.

Figure 1 depicts improvements in CNC and AzBio(Q) scores from preoperative testing to 6- and 12-month post-CI for all subjects by age. The R^2^ values for CNC score improvement at 6 months (R^2^ = 0.0488) and 12 months (R^2^ = 0.041) indicate the status of the contralateral ear (ie, SSD vs AHL) accounted for very little of the variance in improvement. For AzBio(Q), the combined-group linear regression showed minimal explained variance at 6 months (R^2^ = 0.042) and increased explained variance at 12 months (R^2^ = 0.1822), the latter of which appeared to be driven by a single influential data point.

**FIG. 2.**
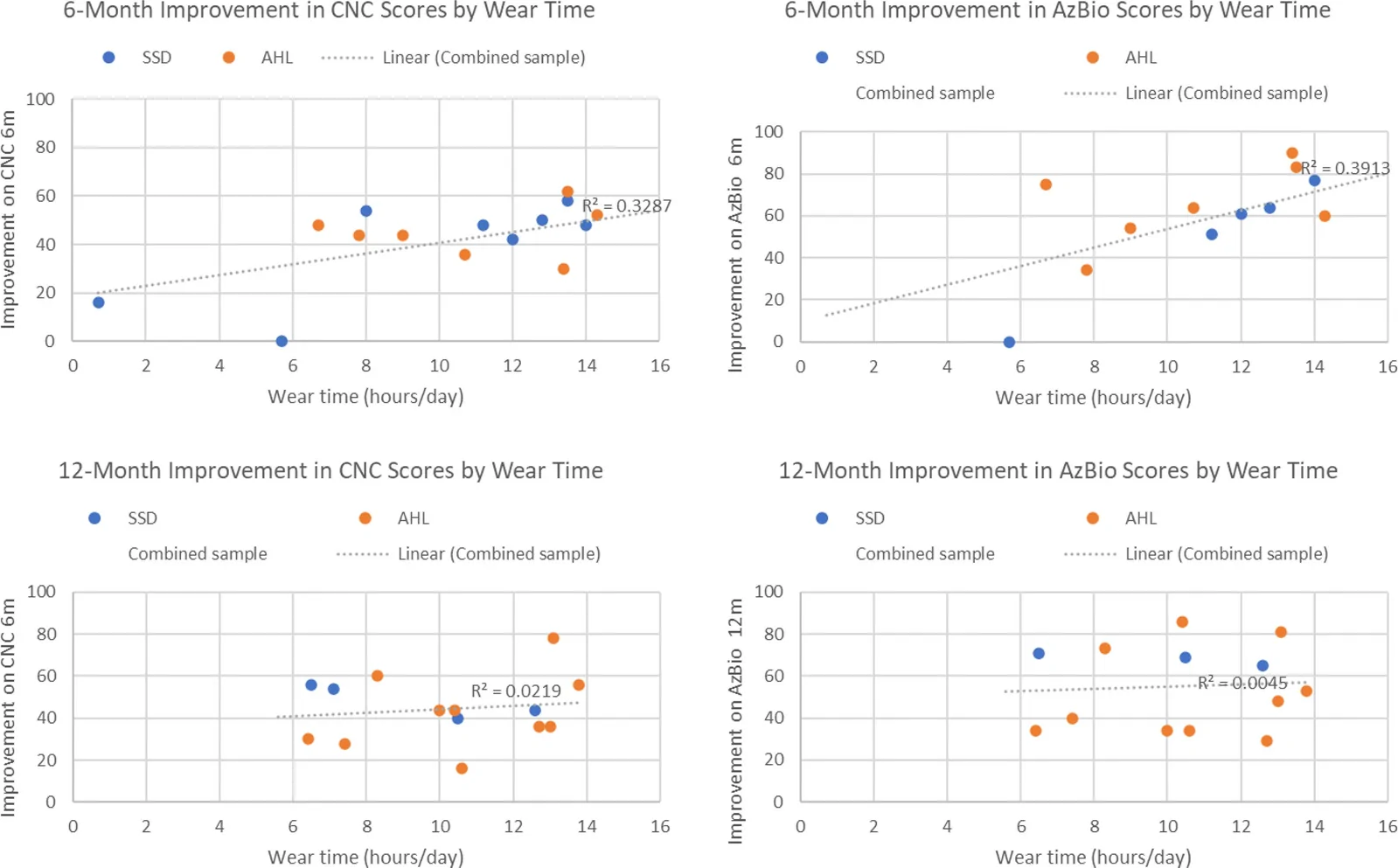
Relationship between average daily device use and speech recognition improvement at 6 and 12 months.

There were no statistically significant differences in average daily device use (hours/day) between the SSD and AHL groups at any post-implantation interval, with both groups approaching or exceeding the recommended target of 10 hours per day (11). Figure 2 depicts the relationship between average daily device use and improvement in speech perception at 6 and 12 months. At 6 months, moderate positive associations were observed for both CNC (R^2^ = 0.3287) and AzBio (R^2^ = 0.3913), indicating a relationship between increased wear time and early improvements in both word- and sentence-level speech recognition. By 12 months, these relationships persisted, but the effect size was smaller (R^2^ = 0.0219 for CNC; R^2^ = 0.0045 for AzBio), likely reflecting the more uniform and consistently high device use among participants rather than a lack of influence of wear time.

## DISCUSSION

The present study examines the safety and efficacy of cochlear implantation in a population of adults ≥65 years of age with SSD and AHL. While published data in this population remain limited given inherent challenges related to CMS coverage restrictions, data from the present study corroborate results from 2 prior publications evaluating outcomes exclusively in older populations. Specifically, Smith et al (5) reported on CI recipients (2 SSD, 10 AHL) treated in a Veterans Affairs Medical Center (mean age 72.6 years, SD 8.8). Significant improvements in the implanted ear were demonstrated for CNC words (from 0% to 42% [interquartile range, IQR: 18–78], z-score:−2.21; *P* = 0.027), CNC phonemes (from 0% to 60.0% [IQR: 36–92], z-score: −2.023; *P* = 0.043), and AzBio sentences in quiet (from 0% to 48% [IQR: 39–80], z-score: −2.521; *P* = 0.012). Mean device use was a robust 9.3 hours per day, supporting significant patient-perceived benefit and approaching the target of 10 hours per day associated with improved outcomes in traditional CI candidates with bilateral hearing loss (11). Both ear-specific speech outcomes and device use are comparable to the findings of the current study, as were the tolerability and perioperative safety between veterans and the current study population.

While both Smith et al (5) and the current study report short- and medium-term follow-up (9–12 months), Johnson et al present longitudinal (5-year) outcomes data on a subgroup of adults >65 years of age with severe-to-profound unilateral hearing loss (UHL) or AHL who were part of a prospective FDA-approved clinical trial for adults (≥18 years of age) (8). Their cohort included 18 adults (mean age: 72 years, SD: 66–79) followed for a total of 5 years beyond the initial study endpoint of 12-month post-activation. Initial (12 months) and long-term (>12 months) results for the full sample (aged 18–79 years, reported in aggregate) substantiated changes to FDA labeling for unilateral and AHL indications, and have been previously reported (12 month (12,13–15); >1 year (16–18)).

In addition to speech perception outcome measures, Johnson et al (8) reported on outcomes for sound localization, quality of life, and tinnitus among older adults >65 years following implantation. For speech perception, improvements in CNC words parallel those seen in the current study and among Veterans (5), and provide valuable evidence of long-term stability in performance (mean; pre-CI: 8%, 1 year: 51%, 5 years: 50%) (7). Johnson et al assessed quality of life (QoL) using the Speech, Spatial, and Qualities of Hearing scale (SSQ) (19), a standardized, disease-specific QoL metric known to be responsive to change in the SSD and AHL population. The significant gain in the spatial hearing subscale from the mean baseline score was maintained out to 5 years post-CI (mean score; pre-CI: 3, 1 year: 6, 5 years: 6). Additionally, the tinnitus handicap inventory (THI), a validated metric of tinnitus severity (20), showed a significant reduction in severity from the mean baseline score, which was maintained in the long-term (mean THI score; pre-CI: 16, 1 year: 4, 5 years: 6). Of note, these values exceed the established minimally clinically important difference of a change score of 7 associated with meaningful improvement on the THI (21), recognizing this benchmark was derived from non–CI-specific tinnitus populations.

The work by Johnson et al (8) also included an objective assessment of spatial hearing in which the target sentence was presented from the front (ie, 0° azimuth) and the masker was either co-located with the target (S_0_N_0_), presented toward the affected ear (S_0_Nci), or towards the contralateral better-hearing ear (S_0_Ncontra). Data from the >65 year-old cohort demonstrated significantly improved masked sentence recognition in the most challenging S_0_Ncontra condition (mean; pre-CI: 5%, 1 year: 22%, 5 years: 41%.) Sound source localization was assessed using an 11-speaker array spanning 180° and reported root-mean-square (RMS) error, with lower values indicating better sound source localization. As with masked sentence recognition, sound source location improved significantly after CI (mean RMS; pre-CI: 76, 1 year: 40, 5 years: 41) (8).

As detailed above, Smith et al and Johnson et al provide unequivocal data on the positive impact cochlear implantation can have for adults >65 years of age with AHL or SSD. Given the small number of true SSD patients in their cohorts (ie, normal or very near normal hearing in the better-hearing ear) (Smith et al, n = 2; Johnson et al, n = 1) (5, 8), each paper combines AHL and SSD into a single group for analysis. In contrast, the current study offers independent analysis of older CI recipients with AHL (n = 31) and SSD (n = 9), providing both group-specific outcome information and a comparison of performance between groups. Performance of the SSD group in the current study compares to that of published aggregate data in patients <65 years of age (22). Additionally, our analysis of ear-specific CNC word scores at 6- and 12-month post-CI suggests similar performance in the implanted ear in both SSD and AHL groups, indicating the status of the contralateral ear accounted for very little of the variance in improvement. This mirrors similar published literature in younger adult populations where objective speech outcomes and subjective measures of quality of life (THI, SSQ, and Hearing Handicap Inventory) were comparable between groups with SSD and AHL (23).

Among traditional candidates with bilateral hearing loss, the impact of age at implantation on CI performance has been the topic of significant research, with evidence confirming older CI recipients demonstrate significant benefit compared with their own preoperative performance (24–26). Less data is available for older adults with AHL, and even less for those with SSD. Patro et al (27) examined a large group of older adults with AHL (n = 188, mean age: 70 years) and found a significant benefit in the CI-only and bimodal conditions as well as a significant improvement on the SSQ compared with preoperative performance. When analyzed by age, they demonstrated a negative correlation between age at implantation and CI-only and bimodal speech performance, but no difference in SSQ measures (27). The relationship between age and CI benefit in adults with AHL and UHL has been examined in various reports on the aforementioned cohort (adults 23–79 years old) (13,16). While post-CI performance exceeded preoperative baseline scores on all objective and subjective measures, they found older adults took longer to demonstrate significant gains in spatial release from masking compared with younger recipients (13,16). Importantly, however, in their groups, the hearing sensitivity of the contralateral ear covaried with age (ie, the average age of the UHL group was younger (14) than the AHL group (13)), making it difficult to determine the true effect of age on post-implantation outcomes. Even with the (comparatively) larger number of SSD patients in the present study, small group size (n = 9) precludes meaningful analysis of age in this group. Once sufficient data has reached widespread publication, future meta-analysis may offer insight into this question by aggregating data from individuals >65 years old across various studies.

Finally, the present study adds to a growing body of literature supporting ear-specific candidacy criteria for CI (23,27). Unlike common insurance policy coverage by private and federal insurers (eg, Veterans Administration), which consider CI for individuals with UHL, current Medicare guidelines require beneficiaries to have significant bilateral hearing loss in order to qualify for cochlear implantation coverage. The longitudinal follow-up of Johnson et al (8) provides insight into the natural history of hearing loss in the “better” contralateral hearing ear among older adults: they found a significant main effect of interval (*F*_(6,70)_ = 12.01, *P* < 0.001) with worsening of high-frequency thresholds of the contralateral ear over the 5-year period. While not surprising, progressive hearing loss of the nonimplanted ear has unique relevance for Medicare recipients who are subjected to long waiting periods before eventually reaching Medicare CI criteria. This delayed intervention may lead to poorer hearing outcomes overall and is differentially borne by older adults at highest risk of hearing loss (28,29). Overall, these data provide support for significant benefit to older CI recipients with UHL/AHL and constitute compelling evidence for reconsideration of current Medicare eligibility requirements.

The present study is limited by lack of standardized subjective measures included in other cohorts, such as QoL measures, SSQ, or evaluation of tinnitus, all of which are known to be positively impacted following cochlear implantation. This retrospective review reports data obtained in routine clinical practice and, as such, does not include experimental conditions designed to assess sound source localization or hearing in noise testing, allowing demonstration of benefits such as spatial release from masking. Additionally, by reporting exclusively CI-only speech perception outcomes, we have not reported performance in everyday listening conditions (which often included bimodal listening for those with AHL). Longer term data and comparison between other rehabilitation options (such as contralateral routing of sound or bone-anchored hearing aids) were not available for this population.

## CONCLUSION

Cochlear implantation in adults ≥65 years old with SSD and AHL is safe and effective, with clear benefits in speech understanding in the CI-only condition in both the short and long-term. Hearing status of the better (contralateral) ear accounted for little of the variance in CNC improvement following suggestion, providing additional evidence for ear-specific candidacy criteria. Revisions to CMS policy to facilitate individual ear consideration would eliminate the inequity in access currently experienced by Medicare beneficiaries and afford them the same options as younger and other insured adults: specifically, the opportunity to consider CI as an option for treatment of SSD or AHL.

## FUNDING SOURCES

None declared.

## CONFLICT OF INTEREST

Daniel M. Zeitler: Consultant—Cochlear Corporation; Surgical Advisory Board, research funding—MED-EL Corporation; CEO—Institute for Cochlear Implant Training. Maura K. Cosetti: Sponsored research- MED-EL, Cochlear Americas; prior advisory board—Cochlear Americas, Acclarent. Nathalie Chouery and Seth R. Schwartz: None.

## DATA AVAILABILITY STATEMENT

The data that support the findings of this study are available from the corresponding author upon reasonable request, subject to institutional and IRB restrictions.

## ETHICS STATEMENT

The study was conducted under the approval of the Icahn School of Medicine at Mount Sinai’s Institutional Review Board STUDY-24-00149 and Virginia Mason Franciscan Health (IRB # 16-112).

## References

[1] Centers for Medicare & Medicaid Services. National Coverage Determination: Cochlear Implantation. Medicare Coverage Database. 2005. Accessed December 2, 2025.

[2] Centers for Medicare & Medicaid Services. National Coverage Determination: Cochlear Implantation. Medicare Coverage Database. 2023. Accessed December 2, 2025.

[3] Medicare Coverage Determination Cochlear Implantation. Indications and limitations of coverage. Available at: https://www.cms.gov/medicare-coverage-database/view/ncd.aspx?ncdid=245&ncdver=3&=. Accessed December 2, 2025.

[4] U.S. Food and Drug Administration. Summary of safety and effectiveness data (SSED): Nucleus 24 Cochlear Implant System (P970051/S205). Published January 10, 2022. Available at: https://www.accessdata.fda.gov/cdrh_docs/pdf/P970051S205B.pdf. Accessed October 10, 2025.

[5] Smith HJ Takkoush S Mendenhall TJ. Hearing benefits of cochlear implantation in older adults with asymmetric hearing loss. Otol Neurotol. 2025;46:515–520. 10.1097/MAO.0000000000004487 40075242

[6] Kay-Rivest E Irace AL Golub JS Svirsky MA. Prevalence of single-sided deafness in the United States. Laryngoscope. 2022;132:1652–1656. 10.1002/lary.29941 PMC9085960 34757636

[7] Chandrasekhar SS Tsai Do BS Schwartz SR. Clinical practice guideline: sudden hearing loss (update). Otolaryngol Head Neck Surg. 2019;161(1_suppl):S1–S45. 10.1177/0194599819859885 31369359

[8] Johnson BR Dillon MT Thompson NJ. Benefits of cochlear implantation for older adults with asymmetric hearing loss. Laryngoscope. 2025;135:352–360. 10.1002/lary.31718 39206702

[9] Peterson GE Lehiste I. Revised CNC lists for auditory tests. J Speech Hear Disord. 1962;27:62–70. 10.1044/jshd.2701.62 14485785

[10] Spahr AJ Dorman MF Litvak LM. Development and validation of the AzBio sentence lists. Ear Hear. 2012;33:112–117. 10.1097/AUD.0b013e31822c2549 PMC4643855 21829134

[11] Lindquist NR Dietrich MS Patro A. Early datalogging predicts cochlear implant performance: building a recommendation for daily device usage. Otol Neurotol. 2023;44:e479–e485. 10.1097/MAO.0000000000003917 PMC10361622 37442607

[12] Dillon MT Buss E Rooth MA. Effect of cochlear implantation on quality of life in adults with unilateral hearing loss. Audiol Neurootol. 2017;22:259–271. 10.1159/000484079 29298446

[13] Dillon MT Buss E Rooth MA. Cochlear implantation in cases of asymmetric hearing loss: subjective benefit, word recognition, and spatial hearing. Trends Hear. 2020;24:2331216520945524. 10.1177/2331216520945524 PMC7586262 32808881

[14] Buss E Dillon MT Rooth MA. Effects of cochlear implantation on binaural hearing in adults with unilateral hearing loss. Trends Hear. 2018;22:2331216518771173. 10.1177/2331216518771173 PMC5950506 29732951

[15] Lopez EM Dillon MT Park LR. Influence of cochlear implant use on perceived listening effort in adult and pediatric cases of unilateral and asymmetric hearing loss. Otol Neurotol. 2021;42:e1234–e1241. 10.1097/MAO.0000000000003261 PMC8448920 34224547

[16] Thompson NJ Brown KD Buss E Rooth MA Richter ME Dillon MT. Long-term binaural hearing improvements for cochlear implant users with asymmetric hearing loss. Laryngoscope. 2023;133:1480–1485. 10.1002/lary.30368 36053850

[17] Thompson NJ Lopez EM Dillon MT. Cochlear implantation for unilateral and asymmetric hearing loss: long-term subjective benefit. Laryngoscope. 2023;133:2792–2797. 10.1002/lary.30608 36757052

[18] Thompson NJ Dillon MT Buss E. Long-term improvement in localization for cochlear implant users with single-sided deafness. Laryngoscope. 2022;132:2453–2458. 10.1002/lary.30065 PMC9514235 35174886

[19] Gatehouse S Noble W. The speech, spatial and qualities of hearing scale (SSQ). Int J Audiol. 2004;43:85–99. 10.1080/14992020400050014 PMC5593096 15035561

[20] Newman CW Jacobson GP Spitzer JB. Development of the tinnitus handicap inventory. Arch Otolaryngol Head Neck Surg. 1996;122:143–148. 10.1001/archotol.1996.01890140029007 8630207

[21] Zeman F Koller M Figueiredo R. Tinnitus handicap inventory for evaluating treatment effects: which changes are clinically relevant? Otolaryngol Head Neck Surg. 2011;145:282–287. 10.1177/0194599811403882 21493265

[22] Oh SJ Mavrommatis MA Fan CJ. Cochlear implantation in adults with single-sided deafness: a systematic review and meta-analysis. Otolaryngol Head Neck Surg. 2023;168:131–142. 10.1177/01945998221083283 35230924

[23] Sydlowski SA Farrokhian N Carrozza M Jamis C Woodson E. (Even Off-Label) cochlear implantation in single-sided deafness and asymmetric hearing loss results in measurable objective and subjective benefit. Otol Neurotol. 2022;43:e895–e902. 10.1097/MAO.0000000000003623 35970168

[24] Carlson ML Breen JT Gifford RH. Cochlear implantation in the octogenarian and nonagenarian. Otol Neurotol. 2010;31:1343–1349. 10.1097/MAO.0b013e3181edb69d 20729782

[25] Cosetti MK Lalwani AK. Is cochlear implantation safe and effective in the elderly? Laryngoscope. 2015;125:1279–1281. 10.1002/lary.25055 25423907

[26] Murr AT Canfarotta MW O’Connell BP. Speech recognition as a function of age and listening experience in adult cochlear implant users. Laryngoscope. 2021;131:2106–2111. 10.1002/lary.29663 PMC8363561 34043247

[27] Patro A Lindquist NR Holder JT. Further evidence for individual ear consideration in cochlear implant candidacy evaluation. Otol Neurotol. 2022;43:1033–1040. 10.1097/MAO.0000000000003677 PMC9481725 36075098

[28] Barnes JH Yin LX Marinelli JP Carlson ML. Audiometric profile of cochlear implant recipients demonstrates need for revising insurance coverage. Laryngoscope. 2020;131:E20–E07-E. 10.1002/lary.29334 33347621

[29] Miller SE Anderson C Manning J Schafer E. Insurance payer status predicts postoperative speech outcomes in adult cochlear implant recipients. J Am Acad Audiol. 2020;31:666–673. 10.1055/s-0040-1717137 33225433

